# Optimization for enhanced ecofriendly decolorization and detoxification of Reactive Blue160 textile dye by *Bacillus subtilis*

**DOI:** 10.1016/j.btre.2020.e00522

**Published:** 2020-08-31

**Authors:** Selvaraj Barathi, K.N. Aruljothi, Chinnannan Karthik, Indra Arulselvi Padikasan

**Affiliations:** a School of Environmental Science and Engineering, Sun Yat-Sen University, Guangzhou, People's Republic of China; b Department of GeneticEngineering, SRM Institute of Science and Technology, India; c College of Agriculture and Biotechnology, Institute of Crop Science, Zhejiang University, China; d Plant and Microbial Biotechnology Laboratory, Department of Biotechnology, School of Biosciences, Periyar University, Salem, 636 011, Tamil Nadu, India

**Keywords:** Reactive Blue160, Decolorization, *Bacillus subtilis*, Degradation, Phytotoxicity

## Abstract

The bacterial strain capable of decolorization and detoxification of the Reactive Blue 160 dye was isolated from a dye waste disposal site of Tirupur textile industries. The bacterial strain was screened and selected based on its decolorization capability of RB 160dye, which was identified as *Bacillus subtilis* by 16S rRNA sequencing. The strain was tested for the decolorization potential under different physio-chemical experimental conditions (pH, temperature, agitation, non-agitation) and observed a complete decolorization at pH 7 and 35 °C under shaking condition within 48 h of time. The enzymes such as, Lignin peroxidase, azoreductase and NADH-DCI were significantly induced in the strain during the decolorization of RB160 dye. Phytotoxicity and microbial toxicity studies revealed that the decolorized product of RB160 dye is less toxic to the plants and microbes. Thus, our results recommend the prospective use of *B subtilis* in bioremediation of RB160 dye.

## Highlights

• *Bacillus firmus* efficiently degrade and detoxified the textile dye of RB160 even at high concentrations within short duration.
• Induction of azoreductase, Liginperoxides and NADH–DCIP reductase activity in the strain to facilitate dye degradation process at the molecular level.
• The toxicity of the RB160 dye was degraded extremely after the biological treatment at specific condition.
• Bacterially degraded products showed non Phyto-toxicity effect on major agricultural crops.

## 1 Introduction

Synthetic azo dyes are widely used in a lot of industries such as textile dyeing, color photography, paper printing, food, pharmaceutical, cosmetic and leather-based industries as well as components in petroleum products because of their ease and cost-effectiveness in synthesis, firmness with a range of colors as in contrast to that of natural dyes. However, textile industries are using more dyes than other industries and an increase in usage of textile dyes was coupled with higher resistance to environmental degradation leading to pollution problems by textile dye effluents [1,2]. While the dyes are measurable at a very low concentration of 1 mg/L, approximately 300 mg/l is disposed through the effluent from textile industrial processes [3]. Anthraquinone and azo dyes are the two most important classes used for dyeing textile fibers today. Azo dyes cannot be degraded under normal conditions and are not detached from water by conventional wastewater treatment methods due to their complicated structures and xenobiotic nature. A class of azo dyes and their degradation products favor the movement of toxic ground and exterior waters that are contaminated by textile effluents, which may further contaminate the other water sources. The dye pollution lead to the unacceptable coloration of water, obstacle of light diffusion and diminution of diluted oxygen in river ecosystems pose a hazardous risk to the aquatic habitants. Hence, it is very essential to process the textile dye effluents before discharging them into water streams [4,5].

Microorganisms play a vital role in the decolorization and detoxification of textile dyes. Currently, a number of methods have been developed for the management of textile dye effluents, particularly, ecofriendly biological treatment methods have demonstrated the best results at a low cost compared with Physio chemical methods [6]. Several studies were interested in combined aerobic and anaerobic microbial treatments to develop the efficient remediation methods [7]. *P. luteola., P. mirabilis* and *K. rosea* showed promising results for dye degradation under anoxic conditions and several other bacterial species under aerobic conditions [8,9]. Although, the decolorization of reactive dyes was measured by a number of researchers, most of these studies have highlighted only decolorization/degradation of dye, with no reports about the degradation by product [10]. Considering this gap in previous studies, we examined the degradation byproducts of RB 160 dye and evaluated their toxic effects to other organisms in the environment.

In this current study, we focus on the degradation of local textile dye used in Tirupur textile industries. *B.subtilis* bacterial isolates were used in this decolorization and degradation study. We evaluated the decolorization, the biodegradation potential of the bacterial isolate using RB 160 dye and also a mixture of other textile dyes. Enzymes concerned with the degradation were assayed and the metabolites generated after the degradation were analyzed further for its phyto and microbial toxicity.

## 2 Materials and methods

### 2.1 Dye, media and bacterial cultures

The dyes, Reactive blue 160, Reactive red 11, Reactive green 16, Reactive violet 131, Reactive gray 22 were gifted by‘Arthanari’s textile industry, Tamil Nadu, India. The bacteria was cultured and maintained in nutrient broth or nutrient agar (Hi Media Company, Mumbai, India). Bacterial cultures of *Escherichia coli* and *Staphylococcus epidermis* were gifted from the ‘Department of Microbiology, Periyar University’.

### 2.2 Isolation and identification of dye decolorizing bacterial strain

The isolated bacterial strain of *Bacillus subtilis* (KJ162241.1) was identified as dye decolorizing bacterial strain and it was done as previously reported similar properties [11]. The obtained 16S r RNA gene sequence of the strain was compared with the related sequence from different bacterial species, retrieved from the GenBank database using BLAST algorithm. Sequence alignment was carried out in CLUSTALW software [12], and then the phylogenetic tree Neighbor-Joining algorithm was constructed using Bioedit and MEGA 5.1 [13].

### 2.3 Decolorization analysis of RB 160 dye

To examine the efficiency of the bacterial strain to decolorize the azo dyes, the bacterial strain of *bacillus subtilis* was cultured in 3 different 250 mL Erlenmeyer flask containing 100 mL media, after 24 h of incubation, 300, 500, 800 ppm of RB 160 dye was added and kept in shaker till the complete decolorization was achieved. The culture media was examined for decolorization efficiency using the UV-spectrophotometer (Hitachi U 2800, Tokyo, Japan) at 490 nm (λ_max_ for R.B160) at 12 h intervals up to 2 days. Media with dye without bacterial culture was considered as a control. A graph was plotted with time interval on the x-axis and the percentage of absorbance on y-axis (Graphpad, USA Version 6). The decolorization efficiency was expressed as per the following equation [14].

Decolourization (%) = (Initial Absorbance – final Absorbance) / Initial Absorbance x 100

### 2.4 Optimization of physico-chemical parameters

To achieve an optimized condition for the decolorization of azo dyes by the bacterial strain, we used different physio-chemical conditions (temperatures (25−55 °C), pH (5–9), shaking and non-shaking conditions) with different reactive dyes (Reactive Red, Reactive Blue, Reactive Grey and Reactive Violet) in varying concentrations.

### 2.5 Enzyme assay

The bacterial cells were grown in the nutrient broth at 35 °C for 24 h. Cells were harvested by centrifugation at 7000X g for 20 min. The pellet was then suspended in 50 mM potassium phosphate buffer (pH7.4) and sonicated (Sonics-vibracell ultrasonic processor) at 50 amps, 7 strokes each 30 s at 3 min interval at 4 °C. The sonicated cells were centrifuged in cold condition (at 4 °C; 7000 Xg for 20 min) and the supernatant was used as a source of the crude enzyme subsequently for further examination [15]. The azoreductase activity was assessed by modifying the previously explained method by [16]. The modified method reaction mix has a total volume of 2.2 ml with the following components, 20 μM NADH, 50 mM sodium phosphate buffer pH 5.5,152 μM methyl red and 200 μL of enzyme solution. The activity was measured after the addition of NADH, by examining the change in the color intensity at 440 nm. The lignin peroxidase assay was done as described by [17], and the absorbance was measured at 310 nm [18]. The NADH-DCIP movement was determined using previously described method [19] and DCIP reduction was read at 590 nm.

### 2.6 Phytotoxicity and microbial toxicity

Phytotoxicity of the RB 160 dye and its degraded products was assessed using the seed germination assay. The examination was done (at room temperature) on corn and green gram (50 seeds of each) by adding 15 mL of Reactive Blue 160 dye (500 ppm) or their products (from 500 ppm treatment batch), while plain water being a control. Germination (%), length of radical, shoot and root were examined after 7 days [20]. The microbial toxicity of intact RB 160 dye and their degraded products achieved after its degradation (500 ppm) was tested on *B. subtilis, Ecoli, S. epidermis* and the zone of inhibition (diameter in cm) was measured after 24 h of incubation at 37 °C [21].

### 2.7 Statistical analysis

All the assays were carried out in triplicate and the values are represented as mean ± SEM. The statistical analysis was done using one-way ANOVA using Graphpd prism 6.0, with Tukey–Kramer comparison test, p < 0.05 being statistically significant.

## 3 Result and discussion

### 3.1 Identification and phylogenetic positioning of the bacterial isolate

**Fig. 1:**
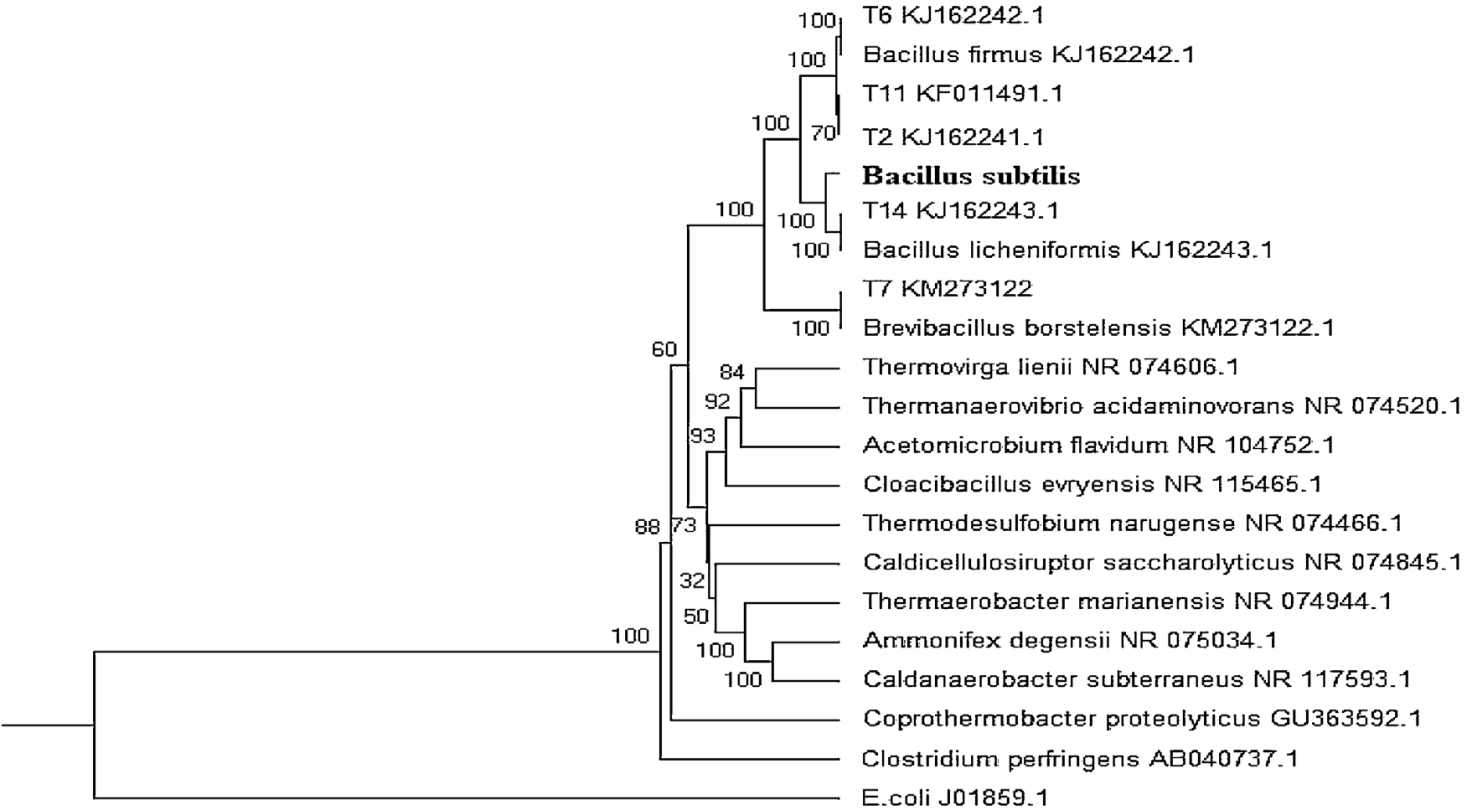
Phylogenetic tree showing the sequential relationship between the dye decolorizing bacterial strain of *Bacillus subtilis*. An algorithm with bootstrap values expressed as percentage of 1000 replicates. Bar 0.05 substitutions per nucleotide position.

The isolated bacterial strain having significant RB160 dye decolorization ability were isolated from dye infected soil samples collected around the Noyyal river basin, Tirupur, India. The 16S rDNA gene sequence revealed that the bacterial isolate T2 as *Bacillus subtilis*. Fig. 1 shows the phylogenetic relationship between the isolated bacterial strain and other related bacteria found in the Genbank database. The homology analysis showed that the strain T2 was in the phylogenetic branch of the *Bacillus* genus, showing maximum similarity with (96 %) *Bacillus subtilis*. *Bacillus* sp. were highly used in the bioremediation methods like to activated sludge method, decolorizing and degrading azo dyes [22,23]. Many other studies have been conducted on this *Bacillussubtilis* sp. to test their bioremediation potential in recent years. For example, disperse yellow 211 and Crystal violet dyes were treated with the Bacillus. sp for decolorization and degradation purposes [24,25].

### 3.2 Effects of initial concentrations of RB 160 dye

**Fig. 2:**
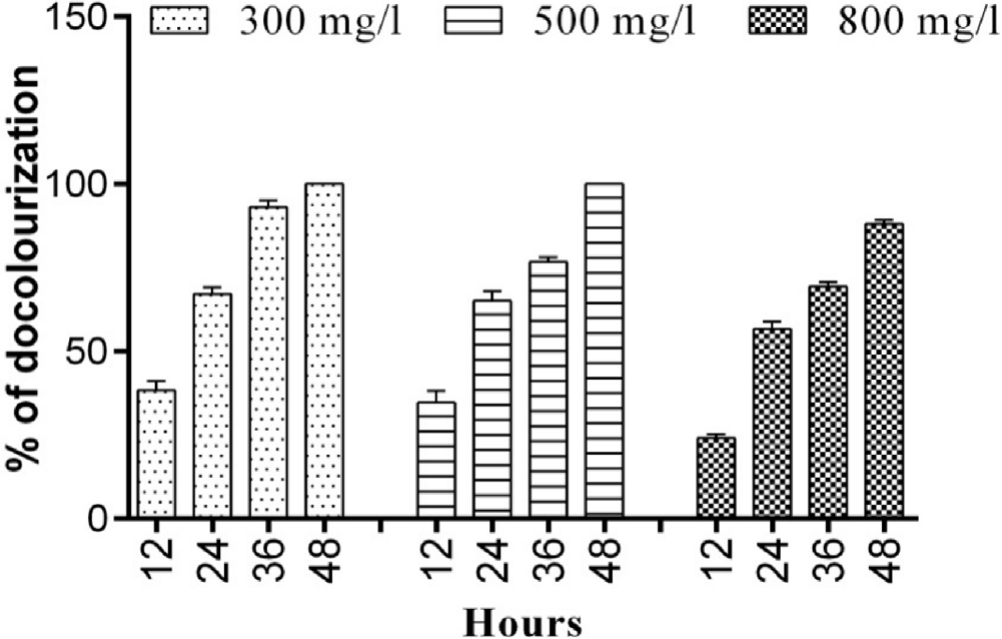
Decolourization capability of the *Bacillus subtilis* strains at different concentration (300, 500 and 800 mg/l) of Reactive Blue 160 dye. (For interpretation of the references to colour in this figure legend, the reader is referred to the web version of this article).

Three different concentrations were randomly selected for (300,500 and 800 ppm) the decolorization treatment. The bacterial strain showed a complete reduction with 300 and 500 mg/l of RB 160 dye after 48 h incubation. However, the bacterial strain was unable to reduce the dye completely at the highest concentration of 800 ppm, in which the maximum decolorization observed was 88 % (Fig. 2). The high concentration of azo dye restrains cell growth and nucleic acid biosynthesis [26], the dye concentration affecting the growth of microorganism is a significant concern in this study. Similar studies that showed microbial degradation of azo dyes, for example, *Pseudomonas aeruginosa* isolated from the tannery effluent site required 24 h for 80 % decolorization of Navitan Fast Blue S5R dye at 200 mg/l initial concentration [27], while, *Pseudomonas luteola* degraded different dyes within 42 h [28]. Moreover, *Pseudomonas fluorescens* has been reported to decolorize 40–80 % of various azo dyes at an initial concentration of 20 mg/l and the decolorization efficiency of *Pseudomonas* sp. in the consortia significantly increased compared to the individual isolates [29]. Previous reports on the dye decolorization had few limitations such as, only low concentration can be decolorized (range of 0.1−0.2 g/l) and a bacterial consortia was required for efficient decolorization, whereas, the outcome of this study affirmed that *B. subtilis* without any aid, can decolorize100 % of the dye at a maximum of 500 g/l initial concentrations.

### 3.3 Effects of temperature

**Fig. 3:**
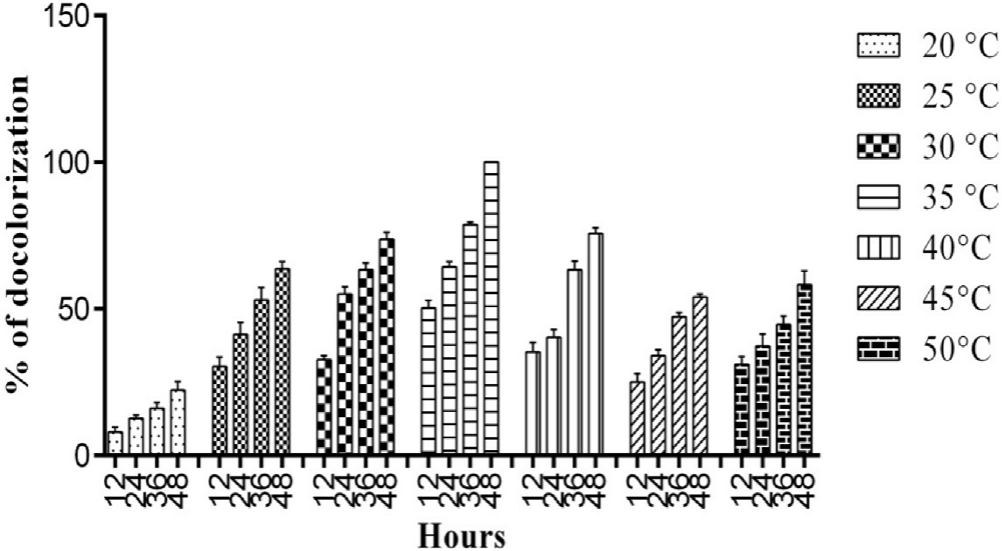
Effect of various temperatures (25 °C-50 °C) on the decolorization of Reactive Blue160 by *Bacillus subtilis.*

Temperature is an important factor associated with the microbial vitality and function, as the microbial cells respond to temperature variations through biochemical alterations [30]. Decolorization assay was performedat 20 °Cto 50 °C temperature range, Fig. 3 shows maximum decolorization was attained (100 % decolorization) at 35 °C. Temperature is the most critical parameter for dye decolorization. The growth of microorganisms at different temperatures is a cumulative activity of a large number of reactions mediated by the enzymes. Therefore a direct relationship exists among the rate of microbial growth, the enzymatic reactions and the temperature [31]. In most cases the growth and activity increase with increased temperature, but it diminishes sharply and rapidly at extreme upper and lower limits of temperature, the same trend was observed in our experiments, bacterial strains showed better decolorization around 25−40 °C. These results are cohesive with the following reports from various studies on dye degradation. Similarly, who reported that decolorization of methyl red was good between 23−37 °C and completely inhibited at 45 °C [32]. Also, some report has maximum decolorization of Red dye ІІ (200 mg/l) at 35−40 °C by *Rhodobectersphaeroides* [33]. However, increase in dye removal efficiency of NBAR12 to 40 °C with maximum activity and thereafter further increase caused a depression of decolorization potential [34]. Similarly, 35 °C have reported to be optimum for maximum decolorization, however, a decrease occurred at 45 °C and at 55 °C very small dye removal was recorded by using *Lactocacilluscasei* [35].

### 3.4 Effects of pH

**Fig. 4:**
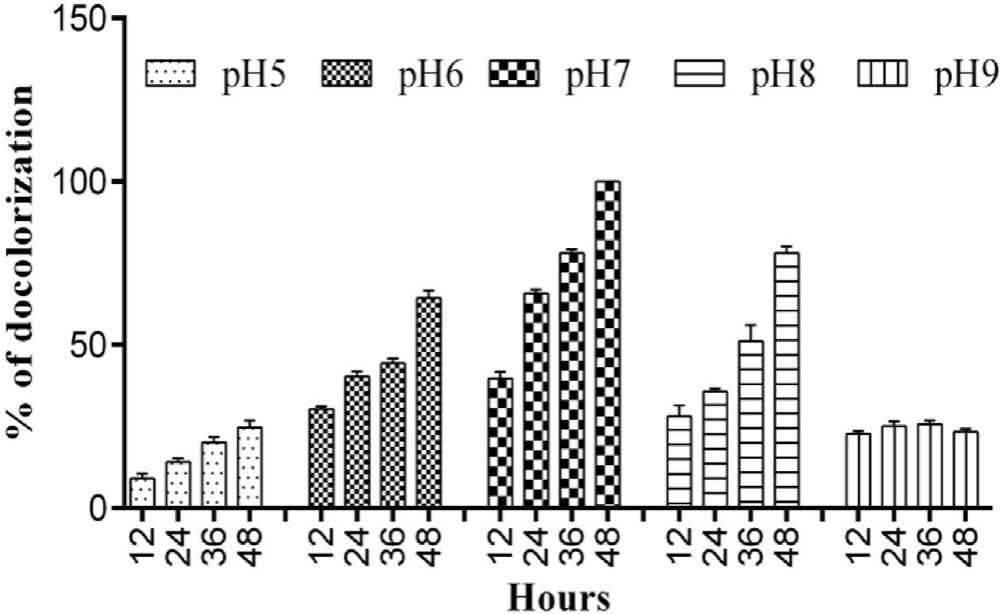
Effect of various pH (5-9) factors on the decolourization of Reactive Blue 160 by *Bacillus subtilis*. (For interpretation of the references to colour in this figure legend, the reader is referred to the web version of this article).

The effect of pH on the decolorization of RB 160by the strain was determined, by testing over a wide range of pH (5.0–9.0). The bacterial strain *Bacillus subtilis* showed the highest dye decolorization at pH 7 (Fig. 4). At this optimum pH, the strain showed 100 % decolorization of RB 160 dye. At pH 8.0, the strains showed 78 % decolorization of RB 160. Whereas at pH 4 and 9, the strain showed the lowest capability of 24 % and 23 % dye decolorization respectively. Similar to several other outcomes, for example, Bhatt et al. have shown that pH 7.0 was most favorable for the decolorization of Reactive Blue 172 by *Pseudomonas aeruginosa* NBAR12. Ref. [36] have reported that neutral pH is more suitable for decolorization of the azo dyes and is appropriate for industrialized applications. Thus, all the remaining experiments were carried out at pH 7.0.

### 3.5 Effect of static and shaking condition on RB 160 decolorization

**Fig. 5:**
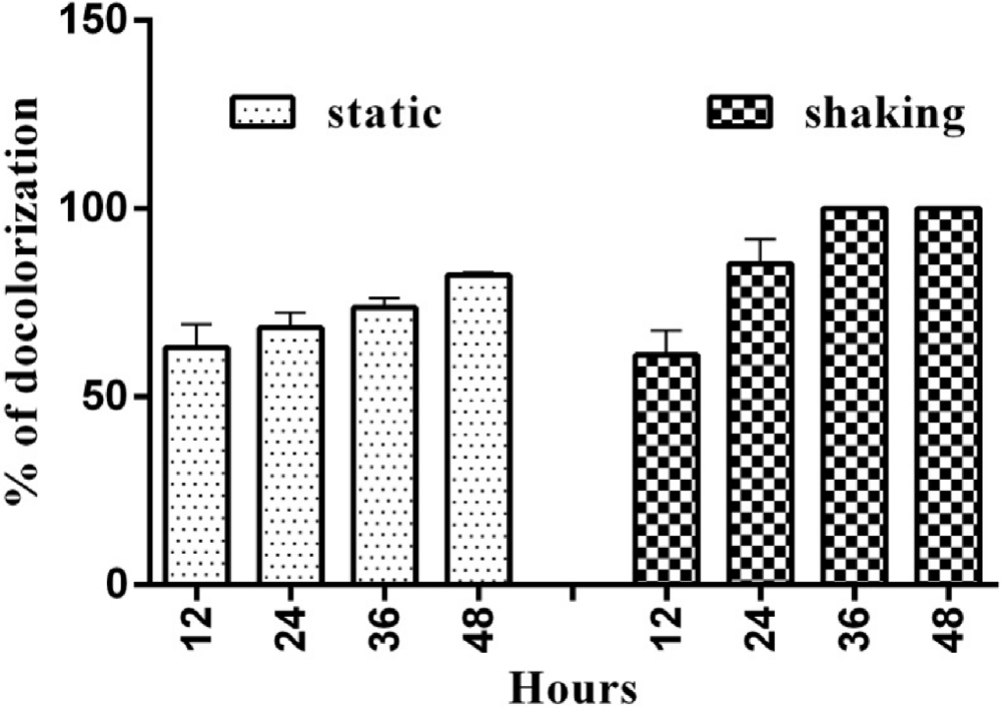
Effect of static and shaking factors on the decolorization of Reactive Blue 160 by *Bacillus subtilis*. (For interpretation of the references to colour in this figure legend, the reader is referred to the web version of this article).

The RB 160 dye decolorization ability of *Bacillus subilis* was studied under shaking and non-shaking conditions. Which is tested bacterial strain showed better decolorization under shaking (100 %) condition compared to the static condition (82 %). While comparing the two conditions, the bacterial strain showed a 100 % dye decolorization at 48 h (Fig. 5). The presence of oxygen normally favors bacterial growth, cell expansion and dye decolorization, and it is one of the mainstream critical factors to be considered in stratifying the remediation methods. Ref. [37] reported the essential role of agitation for the achievement of a high level of decolorization rate by *T. villosa* which are in agreement with our results. Similarly, Coughlin et al. has reported degradation of an azo dye AO7 by an obligate aerobic s*phignomaonas.*sp using dye as an only form of carbon and nitrogen [38]. In contrast, there are few reports suggesting that agitation might lead to inhibition of decolorization due to inhibition of azoreductase enzyme [39].

### 3.6 Decolorization of other Azo dyes by *B. subtilis*

**Fig. 6:**
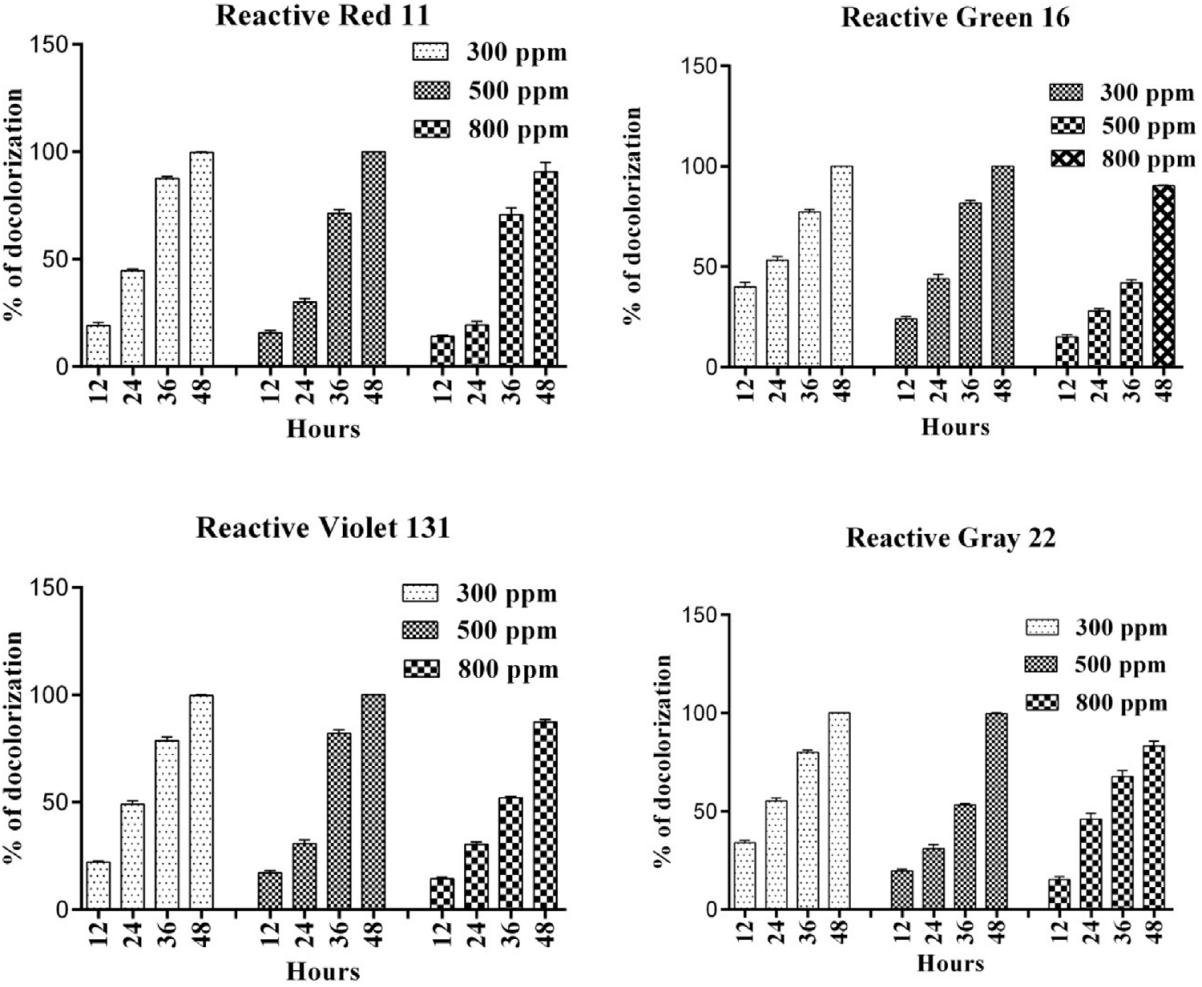
Effect of different concentrations of R. Red 11, R. Green 16, R. Violet 131 and R. Gray 22 on decolorization ability of the *Bacillus subtilis*. (For interpretation of the references to colour in this figure legend, the reader is referred to the web version of this article).

To determine the dye decolorization ability of strain on other kinds azo dyes, it was tested with different concentrations (300, 500 and 800 ppm) of various textile dyes (Reactive Red 11, Reactive Green 16, Reactive Violet 131 and Reactive Gray 22). At 300 and 500 ppm concentrations, the strain *Bacillus subtilis* showed a complete (100 %) decolorization after 48 h incubation. As expected, the dye decolorization ability of the strain was reduced significantly with increasing concentrations of dye. In which, lower decolorization was observed against R.R 11 (56 %) followed by R.G 16 (60 %), R.V 131 (62 %) and R.G 11 (64 %) respectively (Fig. 6). Generally, dyes are highly toxic, non-essential compounds of microorganisms and plants [40]. At higher concentration, textile dyes inhibit the growth of majority wild type bacteria and are tolerated by only few microorganisms. The growth rate of the bacterial strains was studied in the presence (500 ppm) and absence of RB 160. All the bacterial strains showed reduced growth rate when grown in dye amended medium [41]. reported that the viability of *E. coli* cells decreased in the existence of dye. This may be due to the inhibition of the cell division or due to the dye toxicity. Furthermore, the decreased growth at higher concentrations of dyes is likely connected to the modification of genetic material and changed the metabolism and physiological reactions of bacteria. It has been proved that, textile dyes increase generation time and decrease the cell division rate, as the cells take more time to divide during stress conditions [42].

### 3.7 Decolourization mechanism

To decipher the decolorization mechanism, we analyzed the activity of azoreductase, lignin peroxidase, and NADH-DCIP reductase enzymes, which were reported to be involved in the dye decolrization process. The significant incitement in the activity of these enzymes demonstrated their involvement in the dye decolorization (Table 1). Azoreductase is an important enzyme associated with azo dye degrading microorganisms that catalyze the reductive cleavage of the reactive azo bond, which has been eminent in numerous microbes (*P. luteola, Pseudomonas KF46, Bacillus* sp. *strain OY1-, S. aureus P. aeruginosa*). Overall, these azo reductases showed an improved capacity to degrade azo dyes than entire cells. Other reports have shown that, *Bacillus subtilis* bacteria can produce the lignin peroxidase, which has the capacity to degrade polymeric dyes from the dyeing industry and paper brightening through oxidative cleavage [43]. Indeed, reported that NADH-DCIP reductase was enhanced during azo dye decolorization using a consortium of bacteria (Proteus vulgaris NCIM-2027 and Micrococcus glutamicus NCIM-2168).

**Table 1:** Intracellular enzyme activities in of *Bacillus subtilis* cells in induced state (at 12 h and 24 h during decolorization) compared to cells in control (activity Units??).

Enzymes	Control	12 h	24 h
Azoreductase	4.43 ± 0.16	8.81 ± 0.02***	22.4 ± 0.16***
Lignin Peroxidase	0.85 ± 0.23	1.31 ± 0.02**	2.62 ± 0.5*
NADH-DCIP	24.32 ± 1.20	22.60 ± 0.016	25.54 ± 0.008
reductase			

Values are mean of three experiments ± SEM. Significantly different from control (0 h) cells at *P* < 0.05,by one-way ANOVA with Tukey–Kramer comparison test.

^a^Enzyme unit’s min^−1^ mgprotein^−1^.

^b^M of Reactive Blue160 reduced min^−1^ mgprotein^−1^.

^c^g of DCIP reduced min^−1^ mgprotein^−1^.

### 3.8 Phytotoxicity and microbial toxicity studies

**S**eed germination and plant growth bioassays are the most widely used methods to evaluate the phytotoxicity of toxicants [44]. Discharging of untreated dyeing effluents directly into the nearby rivers may cause serious health and environmental hazards and direct impact on the fertility of the soil. Thus, other dye metabolites, for example, unsulfonated aromatic amines are generally stable in aquatic conditions and are improperly degraded under aerobic or anaerobic wastewater treatment conditions, but are completely degraded by biological treatment method [45,46]. The toxicity of the RB 160 dye was evaluated before and after degradation and the results are summarized in Table 2, which signified that the germination (%) and length of radical, shoot and root of both Corn and green gram seeds was less with RB 160 treatments as compared to degradation product. Thus, phytotoxicity experiments revealed that *Bacillus subtilis* have the potential to detoxify the RB 160 dye. RB 160 and its degraded products were tested for microbial toxicity against *B. firmus*, *Staphylococcus epidermidis and E. coli*. The zone of inhibition was not observed in control or the degraded product, whereas RB160 was showed zone of inhibition around 1.22 ± 0.18 and 1.13 ± 0.18 cm against *B.firmus* and *S.epidermidis,* respectively. In contrast, *E.coli* showed zone of inhibition against to both RB 160 dye and degraded product 1.56 ± 0.08 cm and 0.18 ± 0.12 cm respectively (Table 3). In earlier, Shweta et al. and Lamia Ayed et al. have reported that the microbial degraded product does not show toxic effects against the eco-friendly strains. Therefore, this result suggests that the degraded product of the dye is converted into a non-toxic form.

**Table 2:** Phytotoxicity study of Reactive Blue160 and its degradation product.

Parameter studied	Corn			Green gram		
	DW	RB 160	Degraded products	DW	RB 160	Degraded products
Germination (%)	50	12	50	50	15	48
Radial (cm)	3.167 ± 0.589	2.867 ± 0.405	3.050 ± 0.250	3.30 ± 0.305	2.567 ± 1.802	3.567 ± 0.2
Shoot length(cm)	7.60 ± 0.700	ND	8.050 ± 0.650	16.67 ± 0.23	ND	16.50 ± 0.500
Root length(cm)	12.10 ± 0.802	ND	12.50 ± 0.500	7.80 ± 0.305	ND	7.950 ± 0.050

Values are mean of germinated seeds of two experiments, DW: Distilled Water, SD: Standard Deviation.

**Table 3:** Microbial toxicity study of RB 160 dye before and after biodegradation.

Parameter analyzed	Test Organism	Control	Reactive Dye160	degraded products
	Microbial toxicity (Zone of inhibition ± SDin cm)			
Zone of inhibition	*B. firmus*	NI	1.22 ± 0.18	NI
	*St.coccus epi*	NI	1.13 ± 0.18	NI
	*E. coli*	NI	1.56 ± 0.08	0.18 ± 0.12

SD: Standard Deviation, NI - No Inhibition.

## 4 Conclusion

The capability of *Bacillus subtilis* species to decolorize high concentrations of the wide range of azo dyes suggested that the isolated strain could be considered as a valuable element in biological treatment of industrial dyes. Treatment with RB160 induced azoreductase, Liginperoxides and NADH–DCIP reductase activity in the strain to facilitate dye degradation process at the molecular level. Moreover, *Bacillus sutilis* has the high capability to decolorize the RB160 into non-toxic metabolites, which are validated by phytotoxicity and microbial studies.

## The authors statements

Dr. Selvaraj Barathi performed the experiments and wrote the Materials & Methods also a Results and Discussion section; Dr. K.N. Aruljothi analyzed the statistic and grammer corrections; Dr. Chinnannan Karthik helped the experiments and wrote the Introduction and sections. Dr.Indra Arulselvi padikasan has supervised all above process.

## Declaration of Competing Interest

The authors report no declarations of interest.
